# A critical evaluation, challenges, and future perspectives of using artificial intelligence and emerging technologies in smart classrooms

**DOI:** 10.1186/s40561-023-00231-3

**Published:** 2023-02-06

**Authors:** Eleni Dimitriadou, Andreas Lanitis

**Affiliations:** 1 Visual Media Computing Lab, Department of Multimedia and Graphic Arts, Cyprus University of Technology, Limassol, Cyprus; 2 CYENS Centre of Excellence, Nicosia, Cyprus

**Keywords:** Smart environment, Educational technology, Artificial intelligence, Emerging technologies, Smart classroom

## Abstract

The term "Smart Classroom" has evolved over time and nowadays reflects the technological advancements incorporated in educational spaces. The rapid advances in technology, and the need to create more efficient and creative classes that support both in-class and remote activities, have led to the integration of Artificial Intelligence and smart technologies in smart classes. In this paper we discuss the concept of Artificial Intelligence in Education and present a literature review related to smart classroom technology, with an emphasis on emerging technologies such as AI-related technologies. As part of this survey key technologies related to smart classes used for effective class management that enhance the convenience of classroom environments, the use of different types of smart teaching aids during the educational process and the use of automated performance assessment technologies are presented. Apart from discussing a variety of technological accomplishments in each of the aforementioned areas, the role of AI is discussed, allowing the readers to comprehend the importance of AI in key technologies related to smart classes. Furthermore, through a SWOT analysis, the Strengths, Weaknesses, Opportunities, and Threats of adopting AI in smart classes are presented, while the future perspectives and challenges in utilizing AI-based techniques in smart classes are discussed. This survey targets educators and AI professionals so that the former get informed about the potential, and limitations of AI in education, while the latter can get inspiration from the challenges and peculiarities of educational AI-based systems.

## Introduction

The term “S.M.A.R.T” Classroom stands for Showing, Manageable, Accessible, Real-time Interactive, and Testing (Huang et al., 2019), and refers to a setting where the physical space is infused with carefully constructed digital tools and resources to encourage student connection on various social levels, enhance face-to-face interaction in real-time, and record the collective knowledge of the entire class (Lui & Slotta, 2014). A smart classroom is defined as a combination of several high-end technologies that aim to assist educators and students in optimising their overall leaning experience (Micrea et al., 2021). A Smart classroom combines school education and technology (Li et al., 2019) such as mobile technologies, automatic communication and learning tools, video projectors, cameras, sensors, facial recognition software, and other modules that keep track of a variety of environmental factors (Mircea et al., 2021). The role of teachers in the smart classroom is to enhance students' performance, creative and thinking skills (Palanisamy et al., 2020) while also using new teaching methodologies such as social learning, mobile learning, ubiquitous learning (Chen et al., 2016). Although a smart class combines technology with other elements, such as teaching strategies and classroom models, in this paper we focus our attention on the technological dimension of a smart class.

**Fig. 1:**
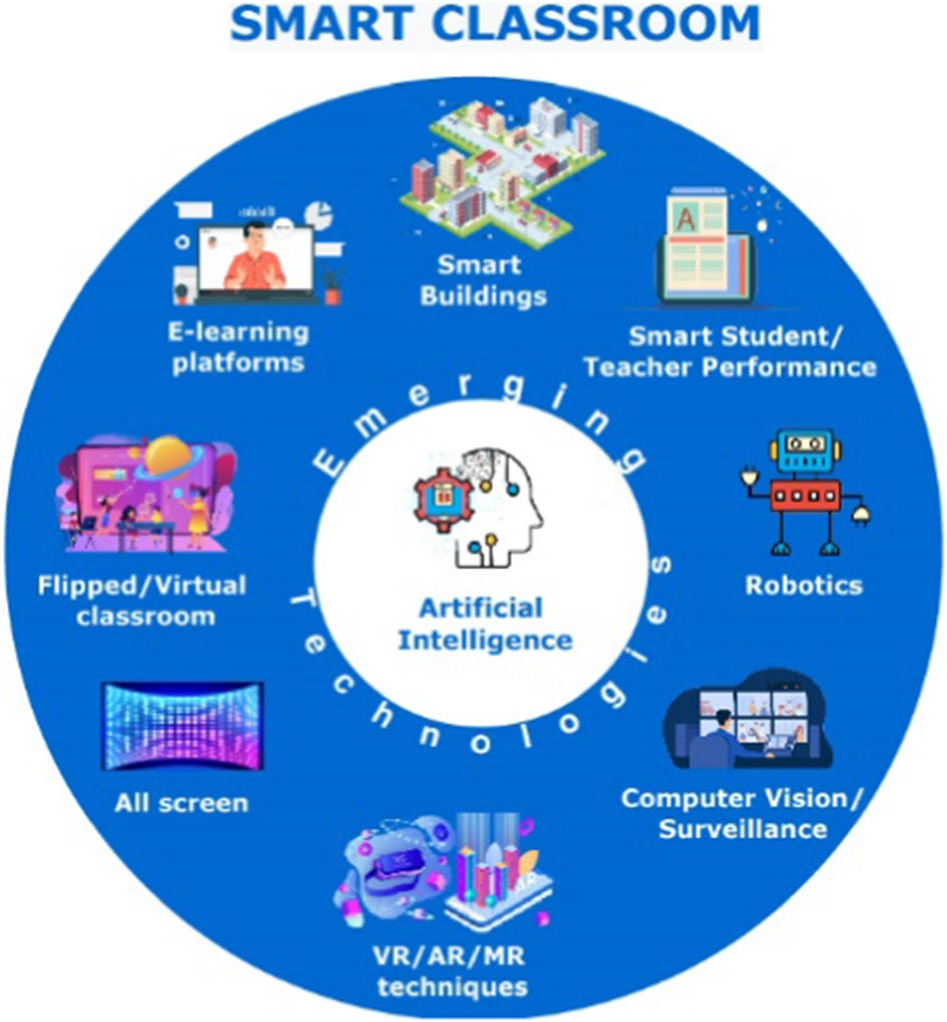
The main technologies encountered in a smart classroom

The introduction of Artificial Intelligence (AI) combined with emerging technologies having the form of interactive, remote, and mobile computing in physical and/ or virtual environments constitutes an evident trend in the development of the concept of smart classroom. Most of the technologies employed in a smart class rely on Artificial Intelligence (AI) that empowers the interactive, adaptive, and smart usage of those technologies during the learning process. In the work presented in this paper, a smart classroom is defined as physical or remote space which integrates emerging technologies (Have et al., 2021) and AI to provide an enhanced learning experience (see Fig. 1).

The term “*Artificial Intelligence*” (AI) was first mentioned by John McCarthyin in 1956 and refers to the ability of computer systems to undertake human tasks (like learning and thinking) that frequently can only be attained through human intelligence (Sadiku et al., 2021). Since the 1970s, the specific field of Artificial Intelligence in Education (AIED) has begun to influence the application of technology to instruction and learning, to improve the learning process, and promote student achievements (Southgate et al., 2019). The aim of AIED is to establish AI-powered systems such as virtual pedagogical agents, AI robots and intelligent systems which allow flexible, engaging and personalised learning as well as to automate daily tasks of teaching (e.g. feedback and assessment) (AlFarsi et al., 2021). The last few years, the topic of AI has been empowered by the groundbreaking technology of deep learning (Sejnowski, 2020) that allowed the successful application of AI to several complex machine learning tasks.

Several surveys related to smart classes appear in the literature. Saini and Goel (2019) focus on technologies related to smart content preparation and distribution, smart student engagement, smart assessment, and smart physical environment. For each pillar Saini and Goel (2019) provide a review of different technologies and techniques used in a smart classroom and provide recommendations for future research directions. While this survey has some similarities to our approach towards the presentation of concepts related to smart classes, in our case we focus our attention on the use of emerging technologies in conjunction with artificial intelligence in smart classes. Furthermore, when compared to the article by Saini and Goel (2019), a wider range of smart technologies are presented. Chen et al. (2020) focus their attention on the use of AI in education. They state that ΑΙ has been extensively used in education in different forms such as computer programs, humanoid robots, web-based chatbots, and online platforms. Despite the wide range of technologies presented by Chen et al. (2020), this survey also includes an extended range of educational technologies coupled with a comprehensive discussion of the advantages and disadvantages of AI in education, that also includes a SWOT analysis. Chen et al. (2022) indicate the usefulness of AI in education, which may be used in the form of intelligent tutoring systems for special education, natural language processing, educational robots, performance prediction, discourse analysis, teaching evaluation, learner emotion detection and personalized learning. Their survey is mainly focused on presenting statistical figures related to the use of AI in education, such as the size of research community per subject, and the most frequent AI terms in literature. More recently, Dimitriadou and Lanitis (2022a) presented a short survey on the topic of the use of AI in smart classes. When compared to this work the current paper provides a more extensive coverage of several key technologies related to smart classes, and in addition it provides a comprehensive discussion of advantages and limitations of using AI in smart classes.

In relation to previous surveys reported in the literature, the main contributions of this survey include the review of the latest technologies and discussion of future directions that could support the creation of a next-generation smart classroom, and the understanding of the use of AI in connection to the technologies used in a smart classroom, allowing the readers to get acquainted with the potential of using AI in smart classes, and the main advantages, disadvantages and potential dangers of using this technology. In the remainder of the survey, we present a literature review for studies related to smart classes and AI in education, and present key smart class technologies related to classroom management, teaching aids, and performance assessment. In Sect. "Impact of smart classroom", we describe the advantages, disadvantages of key technologies related to smart classes. In Sect. "Discussion-future directions", we elaborate on the role of AI in smart classes, followed by a discussion and possible future research directions.

## Key technologies related to smart classes

In this section the key technologies related to smart classes are presented while emphasis is given to the role of AI in the technologies described. The main topics presented are separated in three main categories that refer to class management technologies, teaching aids and performance assessment technologies (see Table 1).

**Table 1:** Taxonomy of key technologies related to smart classes presented

Classroom management	Teaching aids	Performance assessment
Computer Vision-based Surveillance/Security	Robotics	Student performance assessment
Smart Environment	Virtual/Augmented/Mixed Reality	Teacher performance assessment
	E-learning Platforms	
	All Screen	

### Classroom management

The term classroom management refers to the way or approach that a teacher uses to control / manage his / her classroom. Within this scope the management aims to maintain a comfortable and safe teaching environment that contributes to the efficient class delivery. In relation to class management technologies, in this survey we focus on the issues of Computer Vision-based surveillance/security and Smart Environment.

#### Computer vision-based surveillance

Computer Vision techniques in smart classroom are often used for the tasks of Attendance Recognition or Action (Behaviours) Recognition.

##### Attendance recognition

In an attempt to reduce the time need for keeping student attendance records, the process of attendance recognition is often automated based on facial identification technologies (Kawaguchi et al. 2005). For example Chowdhury et al (2020), use a suggested a Convolutional Neural networks (CNN’s) based facial identification system to identify students in a real-time video stream captured by a static camera. Several variations to this technique involve systems that can utilize images captured by moving cameras (Mery et al., 2019), systems that utilize cameras fixed in the entrance of a class (Chintalapati & Raghunadh, 2013), or systems that utilize rotating cameras (Gupta et al., 2018).

##### Action (behaviour) recognition

Human action recognition is a vision-based technique that can identify a complete action performed by a human in a video sequence (Kong & Fu, 2022). The ability to recognize human behavior can be extremely important inside a smart classroom (Wang, 2021) as it enables the recognition of student behavior and emotions allowing the detection of uncomfortable situations for students, such as high anxiety or reduced concertation levels. Smart classrooms with developed AI-powered surveillance system can detect students who are not paying attention in class and alert the teacher (Parambil et al., 2022). In addition, action recognition systems can analyse students’ behaviour during the course and estimate their engagement (Thomas & Jayagopi, 2017). Furthermore, automated action recognition also helps students with special needs by monitoring them and warning teachers for potential episodes, for example in case they have an epilepsy episode (Lau et al., 2014).

Recently, automated methods for the behaviour analysis of the students and their engagement estimation are widely utilized in a classroom. Ngoc Anh et al. (2019), presented a system to monitor the behaviour of students in the classroom. Similarly, Thomas and Jayagopi (2017), used a machine learning algorithm to analyze the students’ engagement in a classroom by analyzing students’ head position, eye gaze direction and facial expressions. Furthermore, Yang and Chen (2011), presented an automatic smart class system which was focused on eye and face detection to determine if the students were active or not.

Previous studies related to recognizing students’ actions in smart classroom include the work of Li et al. (2019) who proposed a new spontaneous actions database that show 15 different student actions. The smart classroom, in this study, included round-tables for students and four cameras, which were fixed on the wall (front and back of classroom), to record the students’ actions from various viewpoints. Recently, Dimitriadou and Lanitis (2022a, 2022b) proposed an action recognition system that recognizes seven actions performed by students attending online courses, which are recognized using CNN architectures. In this case both the images captured and the action recognition process is performed on the personal computer of each student, allowing in that way the application of this method to remote teaching activities.

Ashwin and Guddeti (2020), demonstrated a Hybrid Convolutional Neural Network to analyze students’ body postures, gestures and facial expressions to investigate engagement. Three states of student’s engagement were examined: boredom, engaged and neutral. Rashmi et al. (2021), proposed an automatic system, that monitors the students’ activities, such as the actions of sleeping, eating, using phone, discussion and being engage. The aim of this study is to localize and recognize multiple actions of the students in an image frame.

Similar technologies can be used for recognizing student actions in school yards. For example, suspicious actions, such as student fights, drug delivery, bullying incidents could be detected automatically allowing the prevention of mental and physical health injuries of students. Ye et al. (2018) suggested a strategy to identify occurrences of abuse in the environment of school utilizing motion and audio sensors to evaluate activities and verbal expression. Gutierrez and Troyer (2014) describe a simulator named SimBully to illustrate the impact of public belief and attitudes on abuse occurrences by classmates. Ali et al. (2020) use the YOLOv3 network to recognize student behaviours such as calling, napping, or reading a book indoors or outdoors with the goal to discover any undesirable behaviours.

#### Smart environment

Smart buildings are described as whole structures that use existing technology resources and AI to produce a secure, functioning and friendly setting that utilizes resources wisely and economically (Dryjanski et al., 2020). Sensor technology (Abbasy & Quesada, 2017), the Internet of things (IoT) (Abdel-Basset et al., 2019), external telecommunications and smartphone software technology are commonly used to power advanced technologies in smart classrooms (Wu et al., 2020).

AI plays an important role in the creation and implementation of computer applications in order to successfully manage the administrative issues of a school and assist workers in their everyday tasks. Data collected instantly from sensors and cameras can be utilized for the surveillance of students and in collaboration with AI create a safer environment for students. The use of AI in smart environments and smart classes can increase efficiency and result in high performance of students and teachers as well (Augusto et al., 2009).

### Teaching aids

In a modern smart-classroom, the teaching process is assisted by a plethora of technological means, to maximize the engagement and interaction of students. In this section we provide an overview of these technologies.

#### Robotics

Educational Robots can be ‘Real’ devices (Shiomi et al., 2015; Weibel et al., 2020), or they can be software agents in the form of chatbots (Kollia & Siolas, 2016; Pereira, 2016).

##### ‘Real’ robots

A real robot is a device that can perform actions usually undertaken by humans. Since the introduction of the first robot back in 1980 (Johal et al., 2018) several educational animal-like or human-like robots have been presented to suit different levels of education. Educational robots constitute a subgroup of educational technology, as they are employed to make learning easier, enhance the educational performance of students (Mubin et al., 2013) and assist the students in their active participation in the process of problem solving. The main driving force in introducing robots in the learning process is for creating systems that offer more social interaction and support learning (Timms, 2016). Examples of educational robots used over the years are summarized in Table 2.

**Table 2:** Examples of educational robots used over the years

Year	Robot name	Appearance	Main abilities	References
1980	Logo Turtle	Animal like (turtle)	Walk, draw	Johal et al. (2018)
2000	Asimo	Humanoid Robot	Walk, talk, see	Okita et al. (2009)
2001	Robovie	Humanoid robot	See, hear, speak	Ishiguro et al. (2001)
2004	Nao	Humanoid robot	Walk, dance, speak, see	Loos (2015) and Kennedy et al., (2015)
2006	PaPeRo	Semi-Humanoid	Speak, see, walk	Osada et al. (2006)
2006	Maggie	Semi-Humanoid	Speak, see, dance	Salichs et al. (2006)
2007	Tiro	Humanoid	Walk, talk, see, dance	Han and Kim (2009)
2009	Saya	Semi-Humanoid	Speak, see	Hashimoto et al. (2011)
2020	AV1	Human like	Speak, see	Weibel et al. (2020)
2020	ZenoBot	Human like	Speak, see	Pham et al. (2020)
–	Pet Robots	Animal like	Speak, dance, see	Causo et al. (2016)

Initially, robots were constructed to perform repetitive tasks, without any AI. However, the importance of having intelligent machines that may perform advanced tasks eventually led to the use of a series of sensors that provide information about the environment along with the integration of AI for processing and making decisions based on the information received by the sensors (Brady et al., 2012; West, 2018). Typical sensors used in robotics include microphones, Time-of-flight (ToF) optic sensors and motion detectors (Ben-Ari & Mondada, 2018) used in conjunction with AI algorithms for sensing an environment (Poppinga & Laue, 2019). In addition, ToF cameras on robots can be enhanced by utilizing CCD cameras, and infrared depth cameras. All of the data received by sensors is usually used to train neural network models and educate robots to execute all of their duties, from comprehending a user to effectively reacting (Vega & Cañas, 2019). AI-based functionality incorporated in robots include speech recognition, motion control, computer vision, natural language processing, smart agent technology, movement control, and control for grasping objects.

Students accept and form relationships with robots far more effortlessly because of their interaction, which has been shown to improve psychosocial and physical development (Feil-Seifer & Matarić, 2009), as well as their capacity to interact, which improves the process of learning, makes it more exciting and help learners acquire more knowledge (Han et al., 2005). Social robots, specifically, have been successful in assisting kids with autism in comprehending concepts such as boundaries between individuals and emotional intimacy and in improving independent learning skills (Woo et al., 2021). Robots can become familiar with the personal needs of each student and respond accordingly (Jones & Castellano, 2018). Another important feature of robots is their ability to record students’ expressions and mood changes. Robots not only assist students during their courses, but they are also in advance evaluating their behavior and any emotional disturbances that may suggest despair or stress (Werner-Seidler, 2017). For example, Researchers at MIT have developed a robot called "Teacher bot" that can detect and respond to student emotions to provide personalized feedback and support (Bourguet et al., 2020).

While educational robotics can be extremely useful within a class environment, their use can also extend to teaching activities for students who cannot be physically present be in class supporting in that way remote teaching activities. For example, children that are obliged to stay home or being treated at hospitals may face serious consequences regarding their social development. Thus, missing out long periods of school and social interactions with their peers, due to factors that are beyond the control of children, may result to social isolation and feeling lonely (Helms et al., 2016). To combat the above negative situation, robots may ensure that no classes and time with friends are missed, by enabling children have a continuous connection with their teacher and peers (Soares et al., 2017).”

##### Chatbots

The notion chatbot is a combination of two words: “chat” demonstrating conversation and “bot” standing for robot (Chocarro et al., 2021). Chatbots simulate conversations with human users via the use of instant messaging services. Chatbots, demonstrate high potential as a learning teaching tool for remote students and can offer personal assistance, educational content support (Colace et al., 2018), while they can be used as tutors accompanying the process of learning (Chocarro et al., 2021). Examples of educational chatbots used over the years are summarized in Table 3.

**Table 3:** Examples of educational chatbots used over the years

Year	Chatbot name	Main abilities	Reference or web page
2011	StuddyBuddy	Reply to questions, deliver courses	Tian et al. (2021)
2013	IBM Watson	Reply to questions, distribute material	Morrissey and Kirakowski (2013)
2014	Mongoose Harmony	Reply to questions, enroll, book appointments	https://www.mongooseresearch.com/harmony
2016	Dawebot	Create quiz, reply to questions	Pereira and Juanan (2016)
2016	Botsify	Reply to questions, enroll	Lee et al. (2020)
2017	Nerdy Bot	Reply to questions	Singh et al. (2019)
2019	Amazon QnABot	Reply to questions	Pakanati et al. (2020)
2020	Google Assistant	Reply to questions, play videos and games	Karri and Kumar (2020)

The operation of chatbots is a mix of artificial intelligence and Natural Language Processing (NLP). Natural Language Processing is a branch of artificial intelligence concerned with computers' capacity to grasp written and auditory speech in the same way that humans do (Chowdhury et al., 2003). NLP is consisted of three primary elements strongly correlated with AI, speech recognition and speech generation. All three topics are based on AI approaches, like GAN deep neural networks, in order to enhance the quality of the generated speech (Hsu et al., 2019) and reasoning, that helps bots make predictions and draw conclusions with the aim to respond appropriately in every interaction with a human. Voice interactive interfaces arose primarily as a result of breakthroughs in computer and speech recognition technology (Guttormsen et al., 2011). For example the Amazon Echo is an instance of a voice-interaction-based technology (Teja, 2020). It uses DNN to process any given dataset and translate any language for example and Recurrent Neural Networks (RNN) as a controller.

#### Virtual/augmented/mixed reality

**Fig. 2:**
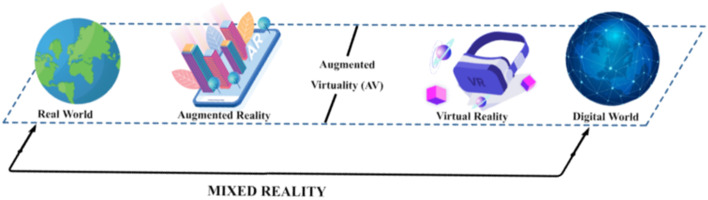
The differences between virtual, augmented and mixed reality

Smart classrooms often incorporate virtual, augmented, and mixed reality as a means of introducing immersive learning experiences. Virtual Reality (VR) concerns the 3D simulation of an imaginary or real environment, that the user can visualize, explore and interact with it (Górski et al., 2016). On the other hand, Augmented Reality (AR) offers an interactive experience to users, by adding virtual information to the physical environment of the students and enabling them to use their whole body as a means of interacting with both virtual and real content (Billinghurst et al., 2015). Mixed Reality (MR) refers to a blending of real-world and virtual/digital world objects which are visualized together on only one display in a coherent space (Kasapakis et al., 2019) (see Fig. 2).

The last few years, the idea of metaverse has risen and is expected to be part of our reality the next few decades. The concept of the metaverse has been around since the 1990s and has gained renewed attention in recent years due to the increasing prevalence of virtual and augmented reality technologies. Metaverse is a parallel digital universe which allows multiple users to emerge into environments that combine both the physical and digital world (Mystakides et al., 2022). Metaverse employs technologies like virtual reality, augmented reality and blockchain to achieve the immersion, as these technologies can achieve multisensory interactions. It provides genuine, physical user interaction and complex interrelationships with virtual objects. In education, Metaverse is not a new concept as several researchers and educators have discussed its implications for learning. Metaverse can be the online space where individuals can meet and socially interact for educational and not only, reasons. So, in the field of education, the metaverse has the potential to revolutionize the way that students learn and interact with educational content. By using virtual reality and other immersive technologies, students can experience educational content in a more interactive and engaging way, which can help to improve retention and understanding. Although, there are some challenges concerning the use of Metaverse in education. Some of them that we are addressing here are data privacy, technology implementation, efficiency, cost, lack of standardization and addiction.

##### Virtual reality in education

VR has been used as an educational tool for numerous subjects (see Table 4). Sobota et al. (2017), states that the two techniques widely used to offer immersive and semi-immersive experience regarding virtual reality in smart classroom are: (a) CAVE (Cave automatic virtual environment) and HMDs (Head-mounted displays) and (b) Interactive school desk. CAVE constitutes a room sized pace including several projection walls where the user is able to move freely in the space and experience their body in immediate interaction with virtual scene and HMDs are suitable devices that offer virtual environment to one user every time. Furthermore, there are different VR accessories which can combine with HMDs and CAVEs, such as gloves, suits or controllers which can offer more exciting experience.

**Table 4:** Examples of educational VR applications used over the years

Year	Author	Topic	Equipment used
2001	Mintz et al. (2001)	Physics/Astronomy	Computer
2002	Knudsen and Naeve (2002)	Mathematics	Head Mounted Displays (HMD)
2008	Adams and Hotrop (2008)	Computer Science	Computer, HMD
2010	Sampaio et al. (2010)	Civil Engineering	Computer
2015	Valdez et al. (2015)	Electrical Engineering	Computer, 3D Max Studio, Vray
2016	Parmar et al. (2016)	STEM	Oculus Rift HMD
2018	Ip et al. (2018)	Autism students	Projection Screens, Camera
2018	Blyth et al. (2018)	Geography	Computer
2019	Alfalah et al. (2019)	Medicine	Projector, Computer, 3D Glasses

The application of VR in education changed some of the previous teaching ideas, but also some of the already existing teaching models (Chen & Tsai, 2012; Gu, 2017). Several studies concluded that VR technologies are more likely to influence the motivation and academic performance of students in a positive way (Ibáñez et al., 2014; Martín-Gutiérrez et al., 2017). Furthermore, Hampel and Dancsházy (2014) argues that the creation of an environment of virtual learning is quite helpful for students, since students are able to acquire knowledge by themselves. Additionally, there is evidence that VR technologies enhance students’ collaborative and communicative skills along with their cognitive and psychomotor skills (Kaufmann & Schmalstieg, 2002; Martín-Gutiérrez et al., 2017; Zhou et al., 2008) whereas VR technologies can be used for the training of the educators as well (Stavroulia et al., 2016).

##### Augmented reality in education

There are different types of augmented display devices in a smart class that include tablets, smartphones, smartboards and different software which enables the creation of augmented scenarios such as Aurasma,^1^ Layar,^2^ Augment^3^ and Aumentaty^4^ (Chamba-Eras & Aguilar, 2017). Oculus Quest, Microsoft HoloLens and Windows Mixed Reality are AR headsets/glasses utilized as augmented display devices. According to Torres et al. (2011), AR in smart classrooms can be used in the following forms: Enlarged book, Virtual models of specific complicated structures, Educational games for the classroom, Virtual models which produce sounds, Magic eyeglasses, Magic mirrors, Magic doors and windows, Navigation support and Cooperative space. Chamba-Eras and Aguilar (2017) state that AR is recommended to compensate various deficiencies that might occur in a smart classroom such as difficulties in doing complicated and dangerous experiments, carrying out actual experiments due to equipment costs, and unavailability of appropriate facilities. Furthermore, the study from Stanford University has shown that students who learn with AR technology demonstrate greater knowledge retention and improved problem-solving skills (Queiroz et al., 2022). The importance of Augmented Reality technology in education has led to its implementation in various topics as shown in Table 5:

**Table 5:** Examples of AR applications used over the years

Year	Author	Topic	Equipment used
2002	Kaufmann and Schmalstieg (2002)	Mathematics	Head Mounted Displays (HMDs), projector, computer
2007	Dünser and Hornecker (2007)	AR books for language education	Camera, Computers, AR markers
2012	Yoon et al. (2012)	STEM and museums	Camera, projector, computer
2014	Ibáñez et al. (2014)	Physics	Tablet
2015	Lu and Liu (2015)	Marine Education	Webcam, AR markers, computer, projector
2017	Alakärppä et al. (2017)	Environment	Android tablets, AR markers
2019	Cai et al. (2019)	Mathematics	Tablet
2020	Kerr and Lawson (2020)	Landscape Architecture	*Google Assistant*
2020	Demitriadou et al. (2020)	Mathematics	Tablet/mobile, AR markers
2021	Reeves et al. (2021)	Biochemistry	Tablet, AR markers
2022	Kim and Shim (2022)	Computer Science and Engineering	Camera, AR markers

##### Mixed reality in education

MR applications in real and smart classroom have many benefits from the perspective of the students in learning and the procedure of obtaining knowledge or skills. According to Dascalu et al. (2014), some benefits by MR to educational uses are: (a) students remain focused on the task at-hand, (b) it is fostered the affective side of learning, (c) computer-based learning gets more human-oriented, and (d) students’ interest and motivation towards learning is enhanced. Furthermore, MR offers immersive and engaging experiences via creative problem solving. MR worlds achieve high levels of immersion through Head Mounted Displays (HDMs), such as Microsoft HoloLens, HTC Vive, Oculus Rift and Magic Leap One, or AjnaLens. Different educational tools were developed to enhance the efficiency of teaching-process such as Virtual Toolkit (Mateu et al., 2015), SMALLable (Tolentino et al., 2009), TIWE Linguistico (Fiore et al., 2014) and Robostage (Chang et al., 2010).

##### The role of AI for VR, AR and MR

The integration of AI in VR/AR applications has the potential to improve its effectiveness, enabling programmers to develop more engaging and fascinating applications (Kaviyaraj & Uma, 2021). The key areas where AI is used in conjunction with VR/AR/MR include the generation of 3D assets, Interaction, Reasoning, Visualization. In the case of AR and MR computer vision capabilities such as pose estimation, object detection, scene labeling and semantic segmentation are used to control content, project an object in the scene, and trigger a spot or occlude objects from the scene (Sahu et al., 2021). Furthermore, AI-based techniques are utilized to generate avatars, digital humanoid characters or complementing users that interact and take decisions immediately according to the gamers' choices, resulting in more engaging experiences (El Beheiry et al., 2019).

#### E-learning platforms

Beetham and Sharpe (2013) state that E-learning platforms (see Table 6) are on-line systems that aim to support synchronous, asynchronous or hybrid learning activities. Within this context, synchronous learning is done in real time, asynchronous learning, is done at a convenient time for the student (Potode & Manjare, 2015), and hybrid learning constitutes of a combination of synchronous and asynchronous learning activities.

**Table 6:** Typical E-learning platforms used in education

Name	Date	Synchronous /Asynchronous	Main Features	Reference
Blackboard Learn	1997	Synchronous	Assignments, tests, communication, grades, announcements	Dobre (2015)
Desire2Learn(D2L) Brightspace	1999	Asynchronous	Documents, communication, feedback, record videos, assignments, data storage, authentication	Moseley and Ajani (2015)
MOODLE	2002	Synchronous and Asynchronous	Course material, assignments, grades, quiz, workshops, communication	Kc Deepak (2017)
Canvas LMS	2011	Asynchronous	Documents, assignments, grades, quiz, communication, analytics, interactive tools	Burrack and Thompson ( 2021)
TalentLMS	2012	Synchronous and Asynchronous	Grades, authentication, communication, gamification, calendar, reports, virtual classes	Agarwa et al. (2019)
Google Classroom	2014	Asynchronous	Documents, email, calendar	Zulkifli and Rozimela (2021)

It has been demonstrated that kids who spend a lot of time on e-learning platforms are more engaged and obtain greater grades because they modify their perception of their schoolwork (Benta et al., 2015). Students seem to be more activated by the way these platforms work and managed to do the assignments they had and create a sense of responsibility to their submissions, as well as to complete some difficult activities (Benta et al., 2014). Students also claimed that this type of environment intrigued them to participate in extra lectures and seminars.

Quite often AI is introduced in E-learning platforms in order to maximize the learning experience of students through the use of adaptive educational systems. The latter can adapt to individual needs and offer support that is tailored to each student, aiming to help students meet their individual goals in the best possible way that fits their personalities and characteristics (Colchester et al., 2017). To this end, adaptive educational systems use the learner profile to diagnose individual characteristics and abilities, the taught model to present the learning material and the instructional model to formulate the content in a dynamic and adaptive way. Recently, a new AI-based e-learning platform called "Edu4AI" has been developed to personalize the curriculum for each student based on their learning style, ability and progress (Geramani et al., 2022). The efficiency of adaptive educational systems proves the capability of AI to assist leaning in multiple ways (Durlach & Lesgold, 2012). Apart from supporting adaptive learning AI is also utilized in other aspects of the learning process. For example, natural language processing algorithms (Chowdhary, 2020) are often used to identify plagiarism and avoid transcribing in assignments submitted by students (Chong et al., 2010). Furthermore, E-learning platforms are used in different classroom models such as Flipped classroom and Virtual classroom.

*Flipped classroom* is an innovative educational method where tasks usually performed in the classroom, like presenting the lecture, are conducted at home, while homework is discussed and performed in class (Akçayır et al., 2018) whereas a *Virtual classroom* is defined as a new educational environment that allows students to attend courses online while also facilitating interaction and collaboration by using the artificial intelligence tools and abilities the platform offers (Rufai et al., 2015). Lo et al. (2017) believe that AI has great potential in the flipped classroom approach, because it may enable the personalization and adaptation of the learning process to the students’ needs. Shan and Liu (2021) suggest a model of Hybrid Teaching of Artificial Intelligence and Flipped Classroom, which combines big data, cloud and online applications to implement comprehensive and individualized learning.

#### All screen

All Screen refers to the ability to project multimedia including audio, photographs, and movies on many screens such as TVs and smartphones. Screen mirroring allows users to access and view the same image or video in two or more screens (Brudy et al., 2019). Screen mirroring is valuable since it might improve the connectivity among a cellphone and a different device, such as smart TVs (Ouyang & Zhou, 2019). Thus, touch gesture input, with virtual buttons on the screen, is a frequent technique for interacting with screens (Ouyang et al., 2021). There are several advantages that all screens may provide to a smart class. To begin with, only wireless screen mirroring makes the connection between instructors' and students' devices straightforward and reliable (Ellern & Buchanan, 2018). Furthermore, practical issues related to the use of projectors, such as connectivity issues, requirement for low-light conditions, and projector noise, are mitigated through the adoption of all screen technology (Sahlström et al., 2019).

### Performance assessment

Student and educator performance assessment and feedback is a highly important education task. Although traditionally performance assessment/prediction has been a quite complex and time-question process, it has been extremely facilitated through automated assessment in a smart classroom (Balfour, 2013).

#### Smart student performance assessment/prediction

Student performance assessment aims on the one hand to inform the teacher about the degree to which students have learnt the content of the lesson and how well they are expected to perform in the future, and on the other, to grade students and provide feedback to them about their performance during the learning process (Saini & Goel, 2019). Traditionally, performance assessment was carried out in a paper or oral format. However, the aforementioned method has many disadvantages, since it is a time-consuming and tiring process, while it results to piles of wasted paper and writing material (Vimal & Kumbharana, 2016).

In a smart classroom dedicated tools can facilitate the performance assessment/prediction through the automation of the assessment. The easiest tool to assess students in a smart classroom is the employment of multiple-choice questions, which allow automated evaluation and feedback, with the aid of an online web server that compares students’ answers with the configured correct answer (Balfour, 2013). An important application of AI in student assessment is plagiarism checking, with Turnitin as a frequently used tool (Ahmed, 2015). Bhatia and Kaur (2021) add an innovative performance assessment/prediction tool based on quantum game theoretic (QGT) decision making. This tool incorporates IoT to gather information and data about students, which are evaluated over a computing platform, aiming to analyze performance and determine the academic enhancement of students. The above methods give the ability to teachers to offer continuous assessment to students, avoiding all the tedious work, while students receive feedback constantly.

Numerous machine learning techniques have been proposed for predicting student performance. Amra and Maghari (2017) propose a system giving predictions regarding the future performance of secondary students based on several attributes. They compared two distinct machine learning algorithms: K-Nearest Neighbors (KNN) and the Naïve Bayes classifier by feeding them with educational data set of secondary schools, collected from the ministry of education in Gaza Strip. Waheed et al. (2020), propose a system to predict the students’ academic performance in a virtual learning environment. Their system used artificial neural networks to classify students in two classes: failure and success, receiving as input data of the assessment performance of 32,593 students provided from an open dataset. Authors made a comparison of the results using baseline methods: logistic regression, support vector machines and Artificial Neural Networks (ANN). The ANN method had the best performance out of the tested models. Warschauer and Grimes (2008), propose the automatic assessment of writing essay assignments with the use of artificial intelligence, using interview data and observations notes as inputs. Students and teachers had positive approach (i.e., student motivation rising, proposing autonomous student activity, constituting a saver of time for teachers).

#### Educator performance assessment

Traditional assessment methods are usually based on the observation of teachers from experts during course time something that can be expensive, not accurate and usually the feedback provided is infrequent and is related to the performance and not on how teachers can enhance their techniques (Archer et al., 2016). To overcome this crucial impediment in teacher development, new technologies are used to produce high quality and meaningful, and continuous automatic feedback for the educators.

Bhatia and Kaur (2021) use IoT systems are used in classes to collect information regarding students and educators to identify their progress. For this purpose, they utilize a Bayesian model and the collected data are assessed through a fog-cloud computing device over time for both students’ and educators’ performance. Jensen et al. (2020) devised a method for teachers to effortlessly audiotape the conversations and lectures in a classroom. They utilized voice recognition and Machine Learning (ML) algorithms to provide generalized estimations of essential aspects of educator speech. Therefore, they state that actual instructor conversation can be captured and evaluated for automated feedback. Jensen et al. (2021) address the issue of designing a framework for automatic educator feedback, that necessitates several considerations about data harvesting processes, automatic assessment and the way feedback is displayed. For this purpose, they employ machine learning techniques, such as Random Forest classifiers, and use transfer learning techniques from BERT algorithm for NLP.

## Impact of smart classroom

In this section, based on information derived from the literature, in combination with critical assessment, the impact of smart classroom on the learning process is analyzed while disadvantages of using key technologies related to smart classes and the impact of AI are also discussed.

### Smart environment

The integration of AI systems that process data collected by Internet of Things (IoT) and other sensors, can help monitor the circumstances of the classroom, offering a safe and eco-friendly environment while they can also monitor students and inform teachers in the case of a student misconduct or potential accidents. AI algorithms can be used to optimize lighting and temperature in classrooms based on occupancy, ambient temperature, and other factors. All these tools and systems can contribute to the establishment of a better and safer learning environment. Furthermore, the interaction with the learning material offered by key technologies related to smart classes can help boost students’ learning, information may be retained more easily and self-efficiency may increase (León et al., 2016). The enhanced interactively offered in smart classes help students have an active role in the class delivery process rather than having a passive role that causes loss of concentration and interest. However, the use of technology for teaching and learning may be related to disconnectedness, which is usually expressed as feelings of separation from learning, the curriculum, the peers and the teachers and the learning devices. Disconnection of students may jeopardize the learning outcomes of students because it results in disengagement, decreased student ownership and absence of student agency. For this reason, teachers in smart classrooms should find ways to remove the barriers to meaningful student involvement and encourage their engagement with the school and the learning process (Blessinger & Wankel, 2013).

Lin et al. (2019) suggest that an adjustable smart system can assist students, improve the learning process and foster a considerable quantity of intellectual learners. Smart classrooms and emerging technologies may overcome the problems related to the provision of timely and individualized support to students, using smart applications that respond to students and provide automated feedback immediately, by comparing current and previous student performance and by motivating students (Vimal & Kumbharana, 2016). In addition, Smart classes support the provision of synchronous and asynchronous education while they support both teacher-led and student-centered activities (Beetham & Sharpe, 2013). Moreover, the above combination allows to enrich learning with the provision of extra material, improve and retain knowledge through the students’ longer interaction with the learning subject, the teachers and their peers. At the same time, the style of the traditional classroom that is offered on scheduled dates and time is retained, resulting to keeping students aware and alert. Having this in mind, and knowing the fact that many teachers are not familiar with technology, while some of them focus on the use of usual software such as Word, PowerPoint, etc., the teachers may face difficulties, or they may need technical support for the full utilization of the emerging and artificial intelligence tools. These teachers need appropriate education to support the successful implementation of a smart classroom with new technologies.

### E-learning platforms

E-learning involves the use of the internet as a platform for educational activities, that can include three-dimensional environments or real-time virtual interactions between students and teachers. The e-learning environment is seen as a crucial tool for supporting conventional learning formats and is changing how higher education is provided. E-learning platforms have several benefits, including improving student-centered learning, helping students become more independent while educators take on innovative roles, encouraging educators to be more reflective and methodical in creating better e-learning resources, and ultimately giving students the skills to adapt to a constantly changing technology-driven environment. AI-powered personalization and adaptive learning can be used to adjust the pace and difficulty level of content for individual students. E-learning enables students to receive learning activities from educators at a distance, allows for larger classes, and makes it easier to identify and record behaviors and errors to improve learning activities. Even though face-to-face learning is better for daily discussions and contributes to an active environment (Valdez et al., 2015), this generation of adolescents dislikes traditional classroom education. Although they like to learn at their own pace, they are curious. Because of their addiction to technology, they rely increasingly on online teaching and learning resources. Through collaborative learning, they enjoy sharing their knowledge with their peers and benefiting from the strengths of their competitors (Agarwal et al., 2019).

### Virtual/augmented/mixed reality

Virtual spaces in a smart classroom resemble real places allowing students to have an immersive experience and create real memories. Moreover, seeing, ‘touching’ and hearing involve more senses in the learning process and link the learning subjects in multiple ways. Therefore, enriched presentation of the learning material and better visualization, which resembles reality and involves more senses, enhances students’ experience and learning becomes sustainable (Lui & Slotta, 2014). Furthermore, the student’s motivation is triggered, situated scaffolding is provided and learning is connected with the student’s everyday life (Bower et al., 2014) through an experiential learning process. AI can be used to create more immersive and interactive learning experiences by tracking student movements and adjusting the virtual environment accordingly. Since a smart classroom is equipped with contemporary visualization technologies, which include interactive whiteboards, projectors, all-screen technology, virtual/augmented reality headsets, cameras, and sensors, students can better visualize the content they are taught, enhancing in that way the learning experience. In a smart classroom, students may be immersed in online virtual environments using headsets; as a result, distractions are removed, and the student’s attention is captured. Furthermore, perspective-changing in virtual reality visualizations allows students to become actors rather than just observers, transforming the learning process into a highly experiential experience (Krüger et al., 2019).

### Computer vision based surveillance

Smart classrooms provide real-time video analysis to educators that want to recognize the behavioural participation and behavioural disaffection of their students (Michalsky, 2021). AI-enabled cameras can be used to monitor the classroom for safety and security, as well as to track student attendance and participation*.* The employment of smart tools and applications, such as cameras, plagiarism checking, and recording, combined with the continuous gathering of data, allows teachers to control student attendance and supervise them both in class and during online assessments (Saini & Goel, 2019).

A challenge concerning the use of advanced technological systems is bias in AI. More specifically, there is a concern regarding how fair can AI systems be to every single student despite the personal attributes of a student (i.e., race and gender) (Li et al., 2021). Groups that face discrimination in the community of technology, like female students, might face more severe inequalities if the creation of AI systems doesn’t consider how to mitigate such biases. Technologically advanced key technologies related to smart classes incorporating AI capabilities are threatened by security and privacy issues because they store and process data that contain personal and sensitive information that may be exposed to potential invaders (Manca et al., 2016). Furthermore, sensors used in classes (i.e. camera sensors or microphones) often used as part of smart-class technological tools, are associated with privacy issues.

One of the greatest challenges in introducing key technologies related to smart classes in schools is the overall cost of equipment required for a comprehensive smart-class deployment is high (Saini & Goel, 2019), preventing in that way the widespread use of smart-class technologies. Bearing in mind that the cost does not refer only to the equipment purchase and initial installation, but also to the continuous upgrade and maintenance, the use of smart class technology involves significant running expenses. Also, because all components of a smart class are somehow disconnected from each other, it is not seamless to integrate all technologies under a common framework, and as a result, the task of setting up the equipment can be a lengthy and time-consuming procedure.

### Robotics

Learning about computers, electronics, mechanical engineering, and languages may be interesting thanks to robots. It has been demonstrated that when language acquisition was facilitated by a robot as opposed to audiotapes and books, young children did better on post-learning assessments and displayed greater enthusiasm (Mubin et al., 2013). AI can be used to control and program educational robots that can interact with students and enhance their learning experience. The teacher in smart classroom takes on the role of a facilitator if the robot is the focal point of the learning activity (i.e., used as a teaching tool, as in the case of teaching about robotics). If the robot plays a passive role, the teacher must provide fundamental knowledge (e.g., by using the robot in language classes). In such cases, robotics curriculum implementation and teacher training are crucial. Looking ahead, it is evident that more has to be done to secure teacher support before robots can be completely included into our schools. Teachers were rated less favorably than parents and children in a survey regarding school robots. Teachers need to be reassured that the objective is not to replace them with robots but to give them a teaching tool or aid that can enhance the educational process and inspire students.

### AI in smart classes: A SWOT analysis

Based on the analysis presented in the previous subsections, the advantages of using AI and emerging technologies may also involve risks that may jeopardise the learning efficiency and experience. In this subsection a SWOT analysis of using AI in smart classes is presented as a means of summarizing the potential of using AI in smart classes, along with possible drawbacks. These observations are presented in a SWOT analysis presented in Table 7.

**Table 7:** A SWOT Analysis of AI in smart classes

Strengths	Weaknesses
Continuous environment monitoring through sensors that results in an optimized learning environment Enhanced Interactivity, including immersive experiences Adaptability to individual needs of students On sight/remote/mixed class delivery	No integrated smart class technology offered Equipment Cost Need for student/teacher expertise in using emerging technologies Need for large amounts of data to train systems Separation and disengagement from the learning process. That results in isolated students

Opportunities	Threats
Availability of state-of-the-art equipment at more accessible cost (i.e. interactive screens, cameras, microphones, VR and AR headsets and glasses) External factors, like the COVID-19 pandemic, dictate the use of technology in teaching as a means of supporting remote teaching Trend towards on-line virtual environments (i.e. META, METAVERSE (Mystakides, 2022) in line with smart-class technologies Latest development in AI that results in accurate algorithms, in the form of deep learning. Availability of ‘public’ ML tools (i.e. lobe.ai, that allows not trained individuals to set up and use ML models)	Privacy issues, ethics and GDBR regulations regarding data collection required by smart systems AI systems and large server stations that store data regarding vital research, may be threatened by hackers Teachers tend to avoid or face difficulties using AI systems due to their inadequacy to adapt to new forms of technology and refuse to accept new technologies as a new norm Cheating-based AI tools may give an unfair advantage to students over their classmates during exams and assessments (Abd-Elaal et. al., 2019) Bias in ML systems that may cause unfair student treatment

## Discussion-future directions

In this section we outline the main issues in the dimensions of technology infrastructure, personnel, and data handling that need to be addressed by the research community in order to maximize the impact of AI in enhancing smart classes.

### Technology infrastructure

To enhance the capabilities of a smart classroom it is necessary to integrate all technologies, hence a combination of emerging technologies and AI is essential. A central AI system that can manage the use of different technologies, suggest optimum ways of integrating each technology in specific classes, and provide a comprehensive evaluation of students and the educational process will be a highly desirable feature of future smart classes.

Since the teaching process is a highly dynamic process where educators need to adapt to the changes in student attitudes and overall class requirements, it is important to deploy AI-based systems that continuously monitor the student requirements and adjust to respond to all changes. While this can take the form of reinforcement learning (Liu et al., 2018) dedicated techniques for AI systems that deal with in-class scenarios, need to be devised.

The integration of special technical equipment is usually an issue due to the expensive cost. As a result, it is critical to adopt new low-cost technical equipment that students may use anywhere, at any time. Experts should develop techniques and technologies that can run on personal equipment rather than dedicated machines, for example using smart phones or low-end personal computers. When it comes to AI-based systems that need to be re-trained continuously, efficient training methods that allow the training process to be completed using ordinary computer systems, need to be employed, so that costs associated with the purchase of dedicated equipment or the purchase of computational time, are decreased.

### Personnel

The need to adopt in practice appropriate teacher training programs regarding the use of technology in education has become urgent. Apart from training for using emerging technologies, educators should also receive adequate training for AI related issues, so that they learn how to harness the power of AI systems for the benefit of the education process. Thus, it is imperative that dedicated AI courses for educators are created, so that smart class teachers are well aware of the potential and risks of using AI empowered emerging technologies. Furthermore, dedicated user-friendly tools that will allow educators to train and use Machine Learning modules should be developed.

Classroom overlays for teachers that incorporate grades, special arrangements, and medical and social information are probably among the educational applications of the future. As technology advances, it will be able to alert teachers to students' learning needs and behavioral issues in real-time and provide solutions. Teachers are sometimes untrained to handle the technical challenges that may occur when a device does not function as planned. As a result, for teachers to succeed, there may be a significant amount of assistance required. To prevent the design of learning from being largely the responsibility of computer scientists who have a limited understanding of successful pedagogy, it is essential that educators learn how to integrate technology into their teaching (Krüger et al., 2019). With the recognition that students' abilities can be impacted by their cognitive, motor, and spatial capabilities, technology also makes it easier for teachers to teach content and learning objectives. Students can become more actively involved in the learning process as they develop their motivation and foundational knowledge (Liono et al., 2021).

Empathy is the ability to recognize somebody's emotional reactions and motives, care for them and their sentiments (Srinivasan & González, 2022). It is vital to develop specialized AI systems that take into consideration the unique characteristics of each student, through an empathetic nature. The topic of producing “empathetic” AI systems can open up several research directions.

### Data handling

One of the most crucial issues of the future of smart classes is ethics in the use of data in AI systems (Borenstein & Howard, 2021). It is vital to address the way data are collected and used by those systems in order to avoid the violation of privacy. Regulations regarding the collection of data must be established and adopted by the scientific community. Data can also be encrypted and anonymised, so in case a hack occurs it won’t be feasible to find correlations between provided data and individuals. Within this scope, new methods that guarantee data security, but at the same time allows the access to the necessary information by different stakeholders, within a smart-class needs to be developed.

Bias in AI is an issue that needs be addressed while using advanced technological systems. More precisely, there is worry about how fair AI systems can be to all students, regardless the attributes of each student such as ability level, race, religion, appearance, or gender (Li et al., 2021). Developers must consider all the biases that may rise due to their personal beliefs and eliminate them. In this way, any form of discrimination towards minorities will be alleviated and students will be able to attend education and receive fair feedback compared to their peers. Furthermore, since supervised AI systems often rely on annotated data, techniques that ensure that any form of bias in the annotation process is eliminated, so that the resulting AI systems are not subjected to any kind of discrimination. AI systems should include machine learning techniques with explainable AI to analyze the educational factors that lead to more fair and effective decision making for students since the ML-based black box model is more understandable to educators (Guleria & Sood, 2022).

Using data collection, user profiling, and adaptive learning can be useful in creating a more personalized and effective learning experience, and artificial intelligence can play a role in supporting these efforts. However, it is important to carefully consider the ethical implications of collecting and using data, and to ensure that students' privacy is protected. The use of AI in education can also raise questions about the role of technology in learning and the potential for it to replace human teachers. While AI can certainly be a useful tool for supporting and enhancing education, it is important to consider the limitations of technology and the value of human interaction and guidance in the learning process. Overall, it is necessary to carefully evaluate the potential benefits and risks of using AI and other technology in education, and to strike a balance between the use of technology and more traditional teaching methods.

## Conclusions

A range of AI-assisted emerging technologies, that include technologies related to class management, teaching aids and performance assessment have been presented. For each smart class technology presented the role of AI was discussed, allowing the in that way the determination of the role of AI in smart classes. Furthermore, through the analysis of advantages and disadvantages of smart classes, along with a SWOT analysis, the prospects, and trends related to the use of AI on smart classes have been discussed, allowing in that way the definition of several future research directions. The future directions presented can provide motivation to the AI, and educational technology research communities to engage in research activities that aim to deal with the identified challenges. Since the new era of technological advancement and the proliferation of digital devices and applications that are routinely used in everyday life has been integrated in education, there is a continuous need to invest in improving the services offered to students and the further development of AI-based smart classes definitely leads those efforts in the right direction.

Since the concept of smart-classes is continuously enriched through the introduction of requirements and new technologies, in the future we plan to monitor this area and produce updated surveys to reflect future developments and conduct investigations in the area of intelligent learning environment. In addition, in the future we plan to provide specific comparative evaluations of different technologies, so that to quantify the effect of existing technologies and highlight the need for future improvements.

## Abbreviations

AI: ARTIFICIAL intelligence
AIED: Artificial intelligence in education
ANN: Artificial neural networks
AR: Augmented reality
CAVE: Cave automatic virtual environment
HMDs: Head-mounted displays
IoT: Internet of things
ML: Machine learning
MR: Mixed reality
VR: Virtual reality

## Footnotes

1 https://www.aurasma.com.

2 https://www.layar.com/.

3 http://www.augment.com/es/.

4 http://www.aumentaty.com/.
